# Modulation of functional network co-activation pattern dynamics following ketamine treatment in major depression

**DOI:** 10.1162/IMAG.a.936

**Published:** 2025-10-15

**Authors:** Brandon Taraku, Jason S. Nomi, Artemis Zavaliangos-Petropulu, Noor Al-Sharif, Paloma Pfeiffer, Viviane Norris, Shantanu Joshi, Randall Espinoza, Lucina Q. Uddin, Katherine L. Narr

**Affiliations:** Ahmanson-Lovelace Brain Mapping Center, Department of Neurology, Geffen School of Medicine at the University of California, Los Angeles, Los Angeles, CA, United States; Jane and Terry Semel Institute for Neuroscience and Human Behavior, Department of Psychiatry and Biobehavioral Sciences, Geffen School of Medicine at the University of California, Los Angeles, Los Angeles, CA, United States

**Keywords:** ketamine, treatment-resistant depression, dynamic functional connectivity, co-activation patterns, rumination, salience network, central executive network

## Abstract

Ketamine produces fast-acting antidepressant effects in treatment-resistant depression (TRD). Prior studies have shown altered functional dynamics between brain networks in major depression. We thus sought to determine whether functional brain network dynamics are modulated by ketamine therapy in TRD. Participants with TRD (n = 58, mean age = 40.7 years, female = 48.3%) completed resting-state fMRI scans and clinical assessments (mood and rumination) at baseline and 24 h after receiving 4 ketamine infusions (0.5 mg/kg) over 2 weeks. Healthy controls (HC) (n = 56, mean age = 32.8 years, female = 57.1%) received the same assessments at baseline and after 2 weeks in a subsample without treatment. A co-activation pattern (CAP) analysis identified recurring patterns of brain activity across all subjects using *k*-means clustering. Statistical analyses compared CAP metrics including the fraction of time (FT) spent in a brain state, and the transition probability (TP) from one state to another over time and associations with clinical improvement. Follow-up analyses compared HC and TRD at baseline. Six brain state clusters were identified, including patterns resembling the salience (SN), central executive (CEN), visual (VN), default mode (DMN), and somatomotor (SMN) networks. Following ketamine treatment, TRD patients showed decreased FT for the VN (p = 7.4E-04) and increased FT for the CEN state (p = 1.9E-03). For TP metrics, SN-CEN increased (p = 5.8E-04) and SN-VN decreased (p = 3.6E-03). Decreased FT for the SN associated with improved rumination (p = 1.9E-03). At baseline, lower FT for CEN (p = 5.70E-04) and TP for SN-CEN (p = 0.016) and higher TP for SN-VN (p = 2.60E-03) distinguished TRD from HCs. CAP metrics remained stable over time in a subsample of HCs (n = 18). These findings suggest ketamine modulates brain network dynamics between SN, CEN, and VN in TRD, which may normalize dynamic patterns seen in TRD at baseline toward patterns seen in controls. Changes in SN state dynamics may correspond to improvements in ruminative symptoms following ketamine therapy.

## 1 Introduction

Major depressive disorder (MDD) is a highly prevalent psychiatric disorder. However, typical antidepressant treatments fail to elicit adequate response in roughly one-third of patients, which are defined as having treatment-resistant depression (TRD) (Gaynes et al., 2009; Nemeroff, 2007). MDD is characterized by disturbances across a wide range functional brain networks, including the default mode network (DMN), frontoparietal or central executive network (CEN), salience network (SN), reward circuitry, and limbic structures, among other brain regions (Cheng et al., 2016; Connolly et al., 2017; Kaiser et al., 2015; Lawson et al., 2016; Mulders et al., 2015; Nestler, 2015; Hamilton et al., 2015; Price & Drevets, 2010). Disturbances across specific networks likely correspond to MDD’s varied clinical and behavioral symptoms (Gorwood, 2008; Hamilton et al., 2015).

Ketamine, a noncompetitive N-methyl-D-aspartate receptor (NMDAR) antagonist, produces robust and fast-acting antidepressant and anti-suicidal effects in TRD patients at sub-anesthetic doses (Yavi et al., 2022). Further, though a single administration of ketamine is unlikely to produce therapeutic benefits extending beyond a week, repeated ketamine therapy is shown to induce stronger and more lasting antidepressant effects (Kryst et al., 2020; Shiroma et al., 2020). Thus, changes in brain function linked with ketamine’s antidepressant effects are expected to be more pronounced after serial ketamine treatment, when a larger number of patients have responded or reached remission. Prior studies have reported functional changes in depressed patients following ketamine treatment across a range of functional brain networks and regions implicated in MDD (Zavaliangos-Petropulu, Al-Sharif, et al., 2023). However, it remains unclear how ketamine modulates functional brain systems to induce its antidepressant effects.

Although a substantial literature has addressed how differences in brain circuitry relate to different functional domains disrupted in MDD, a majority of studies have focused on examining the static properties of functional brain connections (Chai et al., 2023; Mulders et al., 2015; Spellman & Liston, 2020). Static functional connectivity, which computes the average correlation of blood oxygen level-dependent response (BOLD) between regions across the entire fMRI time series (White & Calhoun, 2019), can provide useful information about the average relationship between nodes in a network. However, it ignores the temporal evolution of co-activation in brain networks that fluctuate in their spatial patterns over short periods of time (Bolton et al., 2020; Hutchison et al., 2013). The more recent fMRI studies that have examined these dynamic properties of the BOLD signal have implicated altered functional dynamics that related to a wide range of psychiatric and behavioral conditions (de Lacy & Calhoun, 2019; Fu et al., 2018; Kaiser et al., 2016; Kupis et al., 2021; Marshall et al., 2020), including in MDD (Kaiser et al., 2016; B. Zhou et al., 2022). Furthermore, one study using imaging features to classify depressed and healthy individuals found that dynamic FC-based features were more accurate in identifying MDD than static FC features (Yan et al., 2020). Despite these recent advances in using brain dynamics to study clinical populations, it is not yet known how fast-acting antidepressant ketamine treatment perturbs the dynamics across functional brain networks.

While different approaches exist for investigating brain network functional dynamics, co-activation pattern (CAP) analysis potentially provides an additional dimension for estimating recurring spatial patterns of brain activity across time (J. E. Chen et al., 2015; X. Liu et al., 2013, 2018). Several prior studies have employed CAP analysis to investigate how resting-state functional dynamics are perturbed in MDD. One such study in adolescent depression found that higher dwell time and persistence in a brain state involving activation of the insula and DMN nodes were associated with greater depressive symptoms and rumination (Kaiser et al., 2019). Another study investigating dynamic network alterations in current and remitted MDD, and healthy controls (HCs) found MDD patients spent less time in a CEN state and showed more transitions from the CEN state to the DMN state irrespective of remission status compared with HCs (C. Liu et al., 2023). Additionally, females with MDD who had experienced early life stress were found to spend more time in a co-active CEN-DMN state and transitioned more frequently from this CEN-DMN state to a prototypical DMN state, compared with HCs (Belleau et al., 2022). Notably, aberrant network dynamics involving the DMN and CEN significantly associated with rumination in both studies (Belleau et al., 2022; C. Liu et al., 2023). Prior findings thus suggest that the pathophysiology of MDD manifests as disruptions in functional dynamics of large-scale brain networks, and particular CAP patterns are signatures of ruminative thought.

To understand how the modulation of altered resting-state functional dynamics reported in MDD might contribute to therapeutic response, we sought to determine how CAP metrics are perturbed by serial ketamine infusions (SKI) in TRD patients and how these changes may relate to improvements in depressive symptoms. Given that several prior studies have found associations between aberrant resting-state brain dynamics and rumination, we also examined associations between CAP changes and associations with rumination specifically. Based on prior cross-sectional studies in MDD, we hypothesized that ketamine would alter the functional dynamics of the DMN, CEN, and SN, and that these changes would relate to improvements in depressive mood and rumination.

## 2 Methods and Materials

### 2.1 Participants

Fifty-eight depressed individuals (average age = 40.7 years, n = 28 female) participated in this naturalistic clinical trial. The data acquired for this investigation formed part of a larger NIMH-supported Connectomes Related to Human Disease project, where subjects were followed prospectively while receiving different fast-acting antidepressant interventions. The ketamine arm of the study was pre-registered in clinicaltrails.gov (NCT02165449). Depressed participants received four serial intravenous ketamine infusions over the course of 2 weeks. Data, which included MRI and clinical assessments, were collected less than a week before the first infusion (baseline) and 24 hours after the last (4th) ketamine infusion.

To be eligible for participation, individuals had to meet criteria for TRD as defined by a lack of response to at least two antidepressant trials of appropriate dosage and duration, and experiencing continuous depression for at least 6 months. Additional eligibility criteria included meeting DSM-5 criteria for major depression (First et al., 2015), and having moderate-to-severe depressive symptoms (Hamilton Depression Rating Scale (HDRS) 17-item ≥ 17; Hamilton, 1960), no history of psychotic reactions to medications, alcohol or drug abuse in the past, and no other physical or clinical contraindications to ketamine. Exclusion criteria encompassed unstable medical or neurological conditions, current substance abuse or dependence (confirmed by laboratory testing), or substance abuse within the past 3 months, current or history of psychosis, schizophrenia, intellectual disability, or other developmental disorder, a diagnosis of dementia, and any contraindications to MRI scanning (such as metal implants or claustrophobia).

**Fig. 1.**
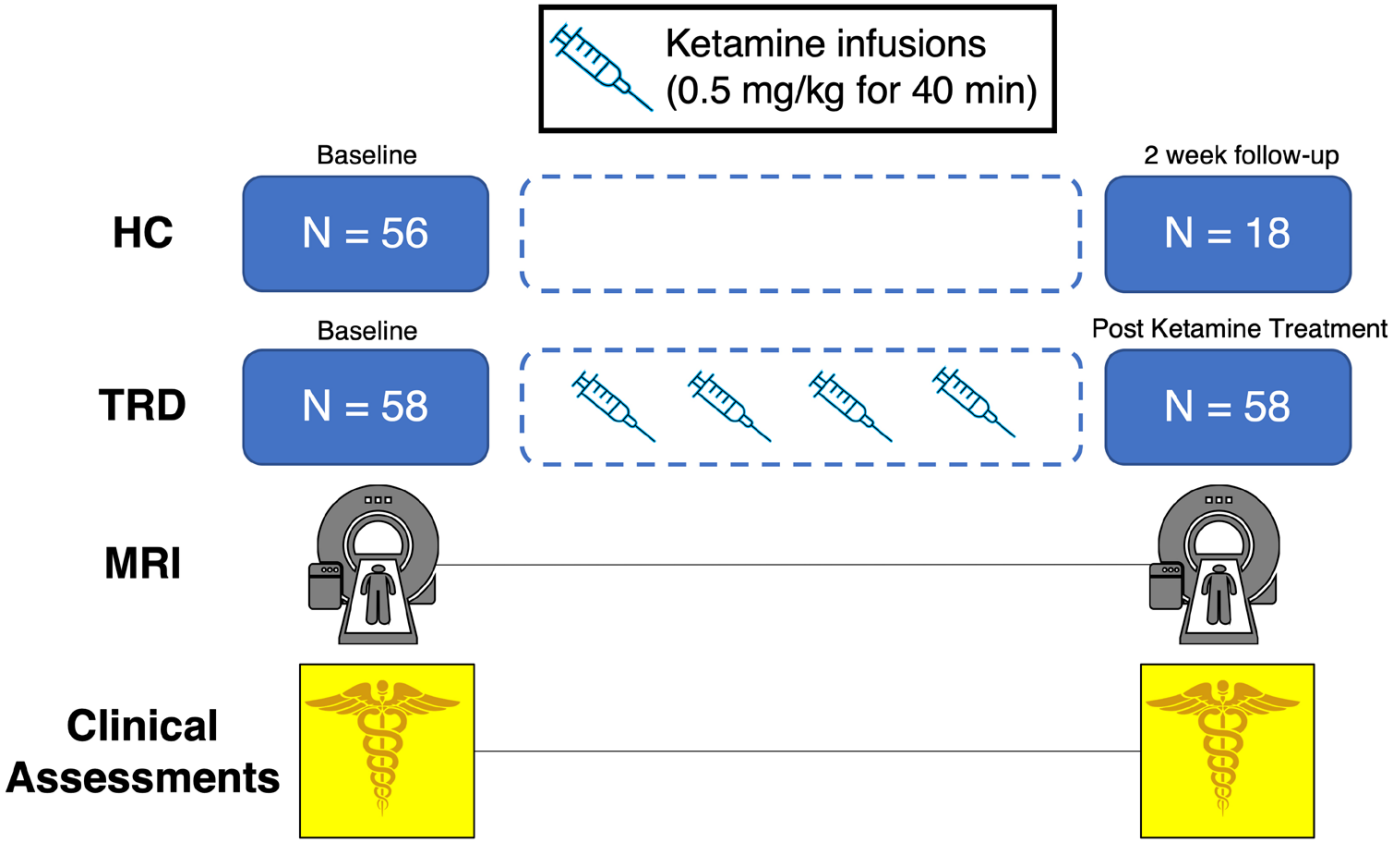
Overview of study design. At each time point, all participants received the same set of MRI scans and clinical assessments. Treatment-resistant depression participants (TRD) (n = 58) were scanned at baseline and after receiving 4 serial ketamine infusions over a period of about 2 weeks. Healthy controls (HC) were scanned at baseline, with a small subset (n = 18) being scanned again after 2 weeks, following a similar time frame to the period of time ketamine was administered to TRD.

In addition, 56 HCs not experiencing depression as confirmed by clinical interview, but otherwise meeting the same exclusion criteria as the depressed participants were assessed at baseline (average age = 32.6, female = 31). A subset of 18 HCs (average age = 29.5, female = 10) received repeated assessments after approximately 2 weeks to mirror the time frame of ketamine treatment in patients. Recruitment of all subjects was conducted in the Los Angeles area through advertisements, clinician referrals, or through clinicaltrials.gov. All participants provided written informed consent in accordance with procedures approved by the UCLA Institutional Review Board (IRB). A graphical overview of our study design is shown in Figure 1.

### 2.2 Ketamine treatment

The administration of ketamine occurred two to three times per week and involved delivering a sub-anesthetic dose (0.5 mg/kg) of racemic ketamine through an intravenous pump, diluted in 60 cc of normal saline. Participants were allowed to continue taking their approved monoaminergic antidepressant medications if the dosage had not changed in the preceding 6 weeks. Benzodiazepines were discontinued more than 72 hours prior to all study visits including scanning sessions.

### 2.3 Clinical assessments

At each time point, overall depression severity was assessed using the 17-item Hamilton Depression Rating Scale (HDRS) (Hamilton, 1960). Remission of depression is defined as an HDRS score of ≤7 post-SKI treatment. Rumination was assessed at each time point using the Rumination Response Scale (RRS) (Treynor, 2003), which includes three sub-scales measuring brooding, reflection, and reappraisal.

### 2.4 Image acquisition

Imaging sequences were acquired at the UCLA Brain Mapping Center using a Siemens 3T Prisma MRI system with a 32-channel head coil, and were identical to those used by the Human Connectome Project (HCP) Lifespan studies for Aging and Development (Harms et al., 2018). Structural sequences included T1-weighted (voxel size (VS) = 0.8 mm isotropic; repetition time (TR) = 2500 ms; echo time (TE) = 1.81:1.79:7.18 ms; inversion time (TI) = 1000 ms; flip angle (34) = 8.0^o^; acquisition time (TA) = 8:22 minutes) and T2-weighted acquisitions (VS=0.8 mm isotropic; TR = 3200 ms; TE = 564 ms; TA = 6:35 minutes), both with real-time motion correction (Tisdall et al., 2016). Resting-state fMRI used a multi-band EPI sequence with opposite phase encoding directions over two runs (VS = 2 mm isotropic; TR = 800 ms; TE = 37 ms, FA = 52^o^, MB accl. factor = 8; phase enc. direction = AP(run1)/PA(run2); TA = 13:22 minutes), along with two sets of spin echo images used for distortion correction (Andersson et al., 2003; Smith et al., 2004). Participants viewed a fixation cross throughout the duration of the resting-state fMRI scan. Additionally, motion censoring was performed for 60 seconds prior to the acquisition of resting-state fMRI scans using the Framewise Integrated Real-time MRI Monitoring (FIRMM) software (Dosenbach et al., 2017), in order to determine the level of motion expected for each subject and provide feedback if excessive motion was present.

### 2.5 Image processing and denoising

Imaging data were preprocessed using the HCP minimal processing pipeline (Glasser et al., 2013). Processing of rsfMRI data included ICA-based denoising using FSL’s multi-run FIX (https://fsl.fmrib.ox.ac.uk/fsl/fslwiki/FIX), and surface alignment using MSMAll. Further fMRI denoising and processing included global signal regression (GSR), band pass filtering [0.01–0.1 Hz], and smoothing with a 4 mm kernel. GSR, which is shown effective at reducing a variety of fMRI artifacts (Ciric et al., 2018; Power et al., 2017) especially motion correlated artifacts in HCP data (Burgess et al., 2016), was performed to mitigate the effects of the global artifacts in the detection of CAP states. Since there remains a lack of consensus over whether GSR should be used in preprocessing, given that it can remove neurologically relevant signals from the brain (X. Liu et al., 2018), we additionally performed CAP analysis with the same protocol described below without using GSR (Supplementary Fig. S4) to determine its effect on our results.

After denoising, rfMR images were parcellated using 400 cortical regions of interest (ROIs) from the Schaefer 7 network atlas (Schaefer et al., 2018), as well as 54 subcortical ROIs from a functional atlas derived using functional connectivity gradients (Tian et al., 2020). Once parcellated, the ROI time series data were z-scored to determine the relative states of activation and deactivation for all regions across the brain. Motion was assessed by examining average framewise displacement (FD) across subjects, and since all subjects moved less than 0.5 mm FD on average, all subjects were retained for analysis. Additionally, the average FD was assessed within each cluster to ensure CAP states were not clustered on the basis of head motion.

### 2.6 Co-activation pattern analysis

CAP analysis was performed in MATLAB by running k-means clustering of fMRI data time points across all subjects and longitudinal scanning sessions using correlation distance to determine the spatial similarity of fMRI time points (1 – rho(X,Y) for data points X and Y) (J. E. Chen et al., 2015; Huang et al., 2020; X. Liu et al., 2013). A maximum of 1,000 iterations until convergence was used with 100 replicates in order to repeat the clustering algorithm with varying initialized cluster centroids, since k-means is highly affected by the initial conditions. By default, MATLAB chooses the replicate with the most optimal clustering score across replicates to ensure robust clustering results. Since k-means requires the number of clusters to be defined, we followed an approach to find the optimal number of clusters by determining the elbow point across a range of clustering scores, as in prior studies from our group and others (Allen et al., 2014; Goodman et al., 2021; Kupis et al., 2021; Marshall et al., 2020). Here, we ran k-means from k = 2 clusters to k = 50 clusters using data from all subjects and time points, and for each clustering solution, the cluster validity index was calculated, defined as ratio of within cluster to between cluster distances (Allen et al., 2014). Once the cluster validity index was computed for all clustering solutions, an L-curve was fitted to the plot of clustering scores using least squares regression to determine the location of the elbow point (Supplementary Fig. S1). Another common approach to find the optimal number of clusters involves computing the Silhouette Score for each cluster (Supplementary Fig. S2).

Next, CAP metrics were computed within each subject’s scanning session, using the optimal number of clusters that were determined to characterize brain dynamics of these states. CAP metrics included (1) the fraction of time spent in a CAP state (FT) (also referred to as the occurrence fraction (C. Liu et al., 2023), dominance (Kaiser et al., 2019), and fractional occupancy (Niu et al., 2025)), defined as the proportion of time points spent in one CAP state relative to all other states, (2) mean dwell time (MDT) or persistence of a CAP, defined as the average amount of TRs one state persists, and (3) the transition probability (TP) from one CAP to another CAP, defined as the number of transitions from states A to B over the number of occurrences of state A, for every pair of states (A, B). TP was modeled as a Markov process, but was calculated after removing repeating CAPs in the time series to control for autocorrelation due to each CAPs persistence (Cornblath et al., 2018; Huang et al., 2020).

Although all data are clustered together to permit meaningful comparisons between CAP metrics across groups and time, we also performed clustering on each individual TRD time points and the HC group to determine the similarity between the clusters formed from each group individually and the clusters formed from all subjects together (Supplementary Fig. S3). While discrepancies may arise between CAP states derived from a sub-group compared with the entire sample, it should be noted that CAP states are cluster centroids defined by the spatial positions of rfMRI time points across a group of subjects. If strong differences in state dynamics (such as the amount of time spent in a CAP state) exist between groups of subjects, then removing the additional groups to identify CAP states from a single group alone would result in a different spatial distribution of rfMRI time points used in clustering, which would likely produce a different set of CAP states. Therefore, it would be hard to disentangle the differences in CAP states between groups and differences in state dynamics within a shared set of CAP states between groups. As such, all primary analyses used the CAP states defined across all groups and focused on examining differences in state dynamics. It should also be noted that most CAP studies focus on examining differences in CAP dynamics rather than the spatial patterns of CAP states, including studies looking across many different clinical groups (Niu et al., 2025).

Furthermore, to determine the effect of cluster number on our results, we performed additional analyses using k_optimal_ + 1 and k_optimal_ - 1 (Supplementary Figs. S5 and S6). Lastly, we performed an analysis looking at changes in static-FC following SKI in TRD to compare the changes in dynamic CAP metrics with traditional static-FC (Supplementary Table S1). Static-FC was computed on the parcellated fMRI time series data, by computing the Pearson correlation coefficient in MATLAB between all pairs of ROI time series, producing a symmetric ROI by ROI correlation matrix.

### 2.7 Statistical analysis

Statistical analyses were performed on the computed CAP metrics and on clinical mood scales. Paired t-tests were used to determine whether significant improvements occurred in symptom scales following SKI. To investigate CAP changes in TRD following SKI, paired t-tests were performed across time for each subject. To determine whether changes in TRD following SKI were significantly associated with improvements in depressive symptoms, Pearson correlations were computed between changes in CAP metrics and percentage change in mood scores ((post-SKI mood score - baseline mood score)/post-SKI mood score) that were significantly improved following ketamine treatment. Statistical analyses of CAP metrics were performed on FT and MDT across all states, and TP was assessed only for states which showed significant effects of FT or MDT, by analyzing transitions from all other states. Multiple comparisons were addressed using Bonferroni correction across all states tested (p < 0.05/6 = p < 0.0083) and all state transitions tested (p < 0.05/10 = p < 0.005) in order to determine the significance level for each set of tests on the CAP data, however, the p-values that are displayed are unadjusted from the tests that are performed. Follow-up analyses were performed on CAP metrics showing significant effects over time in TRD by investigating cross-sectional differences between HC and TRD at baseline, using independent samples t-tests controlling for age and sex, to determine whether changes in TRD moved in the direction of patterns seen in HCs. Secondary analyses focusing on confirming no significant differences existed between HCs and TRD post-SKI, again used independent samples t-tests controlling for age and sex, in order to establish TRD changes as normalizing toward HCs. An additional supplementary analysis was performed using the subsample of longitudinal HCs to test whether longitudinal CAP changes occur in the absence of treatment, such as from arbitrary changes in the scanning environment, in order to ensure the fidelity of longitudinal changes in TRD. Finally, to ensure that results were similar in CAP states derived from TRD patients only, we performed the same set of analyses investigating treatment effects using CAP metrics derived from TRD patients only.

## 3 Results

Significant improvements in HDRS (t = -13.4, p = 4.30E-19), RRS brooding (t = -8.0667, p = 5.33E-11), and RRS reflection (t = -4.617, p = 2.262E-05), but not RRS reappraisal (t = -1.49, p = 0.222) occurred following SKI. Therefore, when looking at correlations between brain changes and improvements in mood, we only focused on HDRS, RRS brooding, and RRS reflection. Demographic information and clinical changes are given in Table 1.

**Table 1.** Demographics and clinical values by group and time point.

	HC	TRD baseline	TRD post-SKI	t/χ2	p-value
Age in years: mean (SD)	32.61 (12)	40.7 (11.3)	-	(HC,TRD) t = 3.70	***0.00034***
Sex: % female	56.36	48.28	-	(HC,TRD) χ2 = 0.035	0.851
HDRS total: mean (SD)	-	19 (4.76)	8.4 (4.6)	(TRD: baseline, post) t = -13.49	***4.30E-19***
RRS brooding: mean (SD)	-	13.4 (3.71)	9.5 (3.13)	(TRD: baseline, post) t = -8.0667	***5.33E-11***
RRS reflection: mean (SD)	-	11.21 (3.8)	9.07 (2.64)	(TRD: baseline, post) t = -4.617	***2.262E-05***
RRS reappraisal: mean (SD)	-	13.9 (4.24)	13.33 (3.37)	(TRD: baseline, post) t = -1.49	0.222
ISCED education variable: mean (SD)	5.96 (1.06)	5.79 (1.2)	-	(HC,TRD) t = -0.776	0.44
Duration of lifetime illness in years: mean (SD)	-	24.78 (16.3)	-	-	-
Current episode in years: mean (SD)	-	8.89 (21.98)	-	-	-
Race (% Asian)	18.18	10.34	-	(HC,TRD) χ2 = 1.427	0.2323
Race (% Black)	20	0	-	(HC,TRD) χ2 = 12.851	***0.0003***
Race (% Hawaiian or Pacific Islander)	1.81	0	-	(HC,TRD) χ2 = 1.064	0.3023
Race (% more than one race)	7.27	1.72	-	(HC,TRD) χ2 = 2.055	0.1517
Race (% other/unknown/not reported)	12.73	6.9	-	(HC,TRD) χ2 = 1.093	0.2958
Race (% White)	40	81.03	-	(HC,TRD) χ2 = 21.433	***3.6E-06***

Bold/Italicized values indicate p < 0.05. TRD: treatment-resistant depression; HC: healthy controls; SD: standard deviation; HDRS: Hamilton Depression Rating Scale; RRS: Rumination Response Scale.

**Fig. 2.**
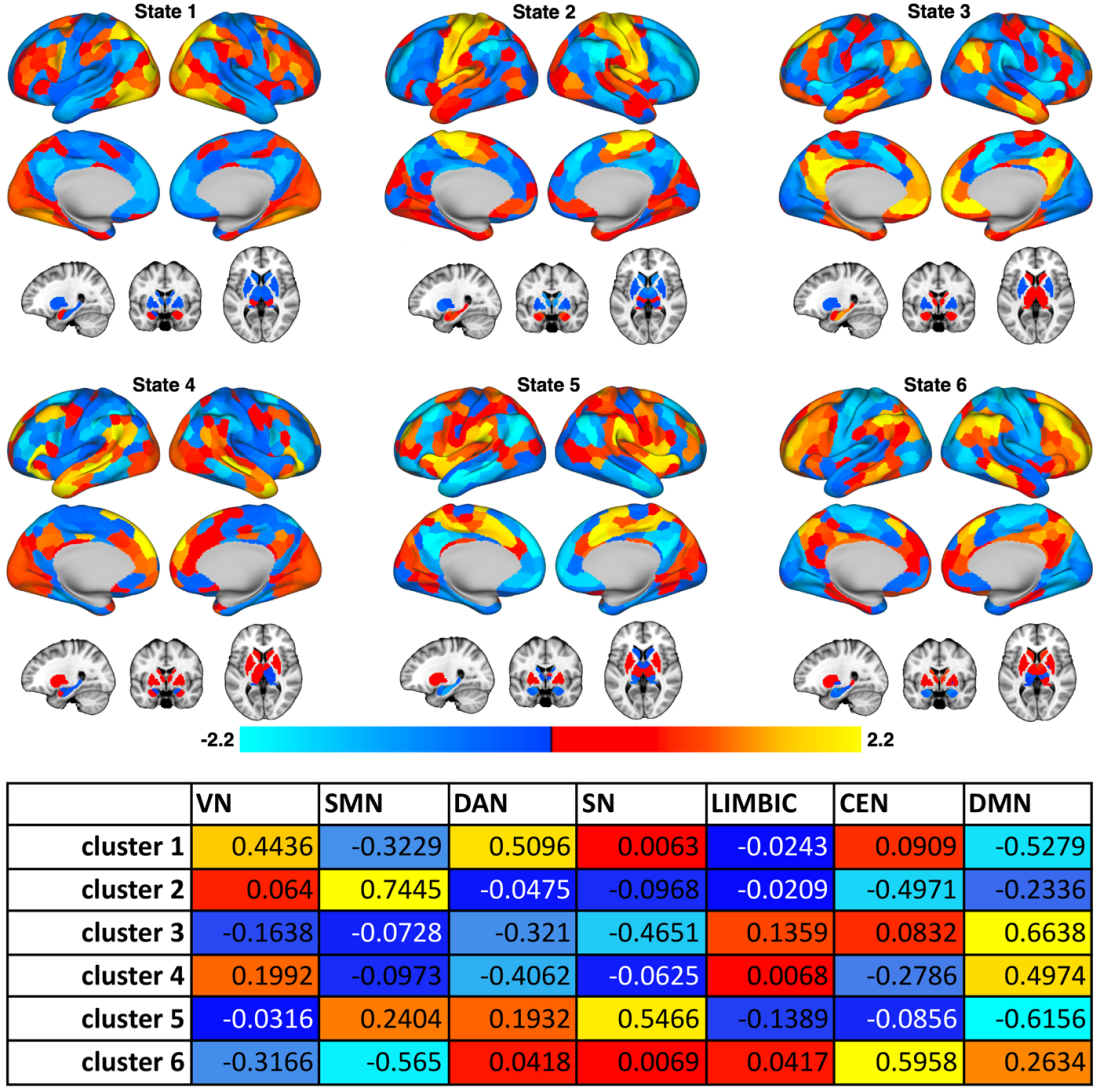
CAP states revealed from clustering fMRI data across all subjects and scan sessions. Custer analyses revealed six states as optimal in representing dynamic brain states. (Top) The centroid of each cluster was projected back onto the brain in CIFTI space to visualize the spatial activation patterns of each brain state, shown on an inflated cortical surface and subcortical volume structures below. Colors are represented as z-scores to show the relative strength of activation or deactivation across brain network nodes. State 1 is characterized by activation in the visual (VN) and dorsal attention (DAN) networks, with moderate activation in the amygdala. State 2 is characterized by activation in the somatomotor network (SMN), with moderate amygdala and hippocampus activation. State 3 is characterized by activation in the canonical default mode network (DMN), amygdala, and hippocampus. State 4 is characterized by activation in ventral and lateral regions of the DMN that are not as strongly represented in state 3, with moderate VN, amygdala, and striatum activation. State 5 is characterized by activation in the salience network (SN), with moderate striatum activation. State 6 is characterized by activation in the central executive network (CEN), weak activation in some medial DMN regions, and moderate activation in the striatum. (Bottom) Each CAP state (rows) was correlated with the set of parcels belonging to each of the seven network states from the Schaefer atlas (columns) in order to quantitatively determine the networks in each CAP state. The correlations show correspondence with the network descriptions based on the visualizations of each CAP state.

Six CAP states were found to represent the optimal number of clusters for all subjects and scan sessions based on the elbow criterion (Fig. 2). These states comprised patterns resembling the visual (VN) and dorsal attention networks (DAN), somatomotor network (SMN), default mode network (DMN), salience network (SN), and central executive network (CEN)—also referred to as the frontoparietal network (FPN) (Uddin et al., 2019). A correlation matrix comparing the similarity across all CAP states, and an analysis comparing the spatial similarity of each subject’s BOLD frame and the associated CAP state across subjects and time points are summarized in Table 2. FD within each cluster and statistics comparing motion across groups are summarized in Table 3. We opted to use these CAP states for further analysis over the ones obtained without GSR, since these states showed higher correspondence with known and clinically relevant resting-state networks. In a separate validation analysis, we found the CAPs states to be similarly represented when only data from TRD patients were included.

**Table 2.** Cluster-wide similarity metrics using six brain state clusters.

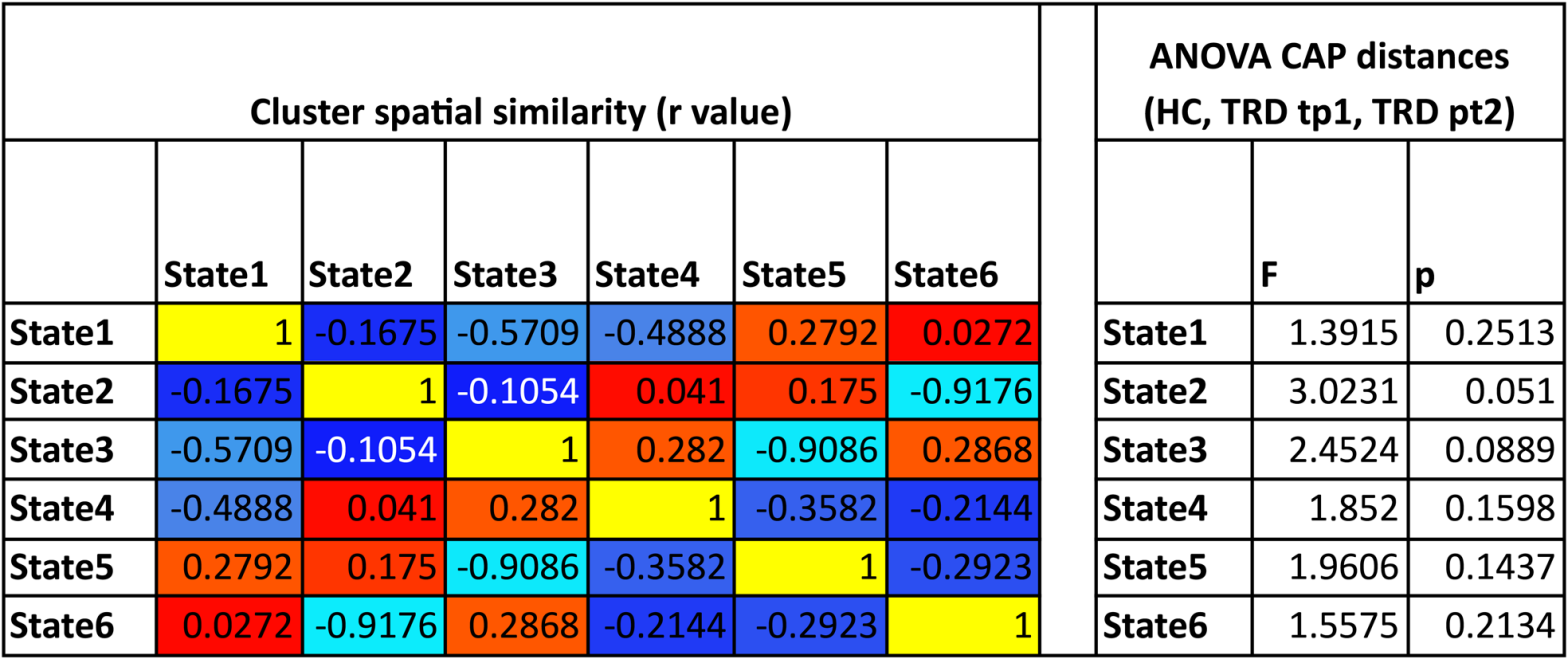

(Left) Pearson correlations were computed between the spatial activation patterns of each cluster centroid to determine the similarity of activation patterns across brain states as a correlation matrix. (Right) To determine whether each subject group was well represented by these sets of clusters, an ANOVA was performed to test for group differences in the spatial similarity between each subject’s fMRI activity patterns and centroid for a given CAP state, similar to an analysis performed in Lee et al. (2024). Results show that no group showed a significant difference in the similarity between their fMRI data and the cluster centroid within any cluster.

**Table 3.** (Left) The average framewise displacement (FD) and its standard deviation were calculated across all frames within each CAP state cluster to determine whether these clusters were driven by head motion.

Cluster motion (FD mm)			ANOVA CAP motion (HC, TRD tp1, TRD pt2)	
	Mean FD	std FD	F	p
State1	0.147	0.1711	1.3915	0.2513
State2	0.1503	0.1713	3.0231	0.051
State3	0.1436	0.1468	2.4524	0.0889
State4	0.1507	0.1717	1.852	0.1598
State5	0.1495	0.1742	1.9606	0.1437
State6	0.1438	0.1471	1.5575	0.2134

All clusters showed low motion on average. (Right) An ANOVA was performed using FD across the three groups (controls, TRD at baseline, and post SKI) within each group to determine whether there were differences in head motion driven by a particular group within any clusters. Results show that no significant differences in head motion were present between groups in any clusters.

**Fig. 3.**
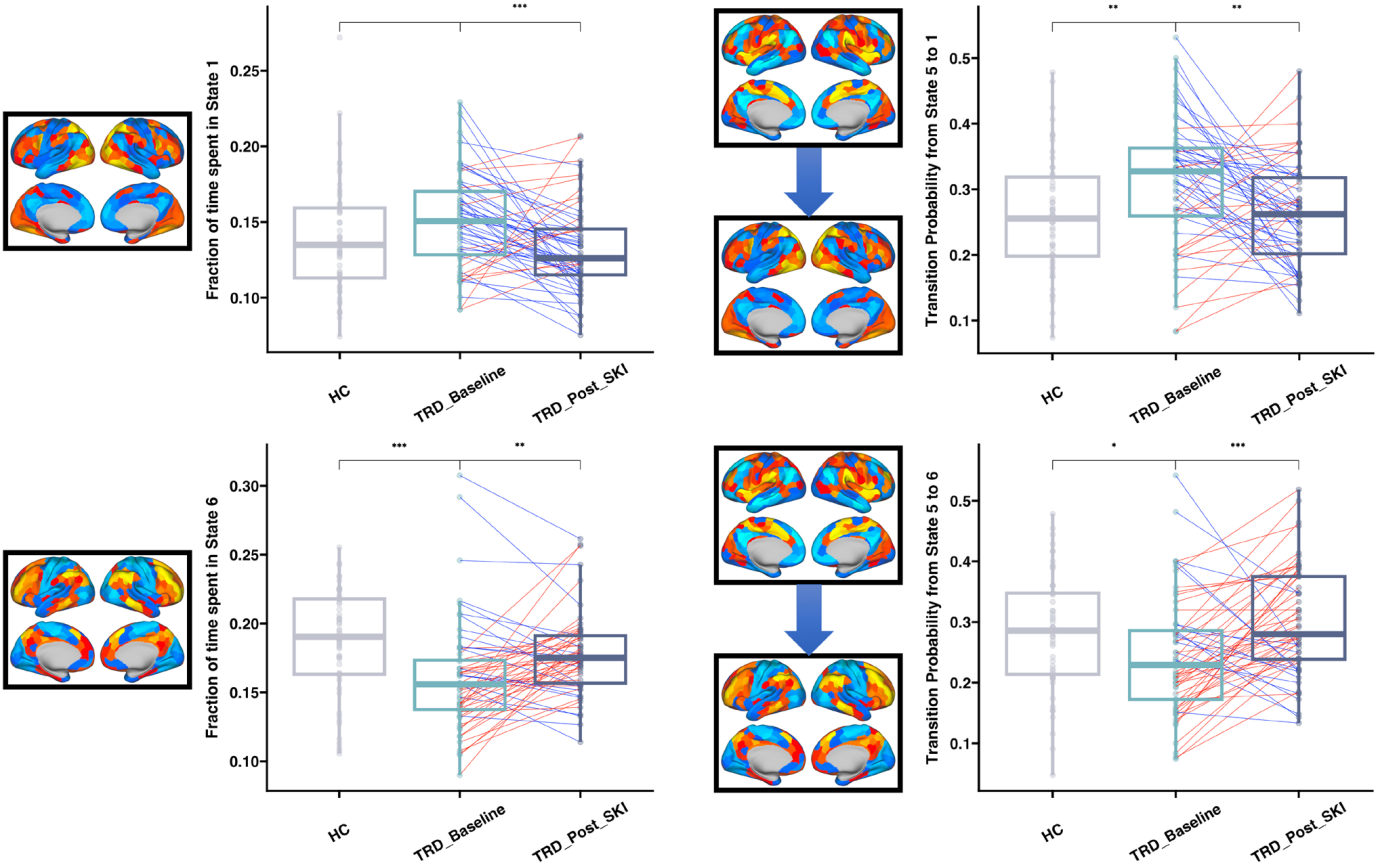
Changes in CAP metrics over time following SKI. Each set of boxplots shows a distribution of values for CAP metrics corresponding to brain states in the TRD sample at baseline (TRD_Basline), following ketamine treatment (TRD_Post_SKI), and in controls (HC). Stars above the boxplots indicate the level of significance for each test performed across the groups (*: p < 0.05, **: p < 0.01, ***: p < 0.001). To the left of each boxplot is a visual representation of the brain states implicated, which include a single brain state or a transition between brain states. Following ketamine treatment, we observed significant decreases in the fraction of time spent in a visual network (VN) state (top left), significant increases in the fraction of time spent in a central executive network (CEN) state (bottom left), significant decreases in the transition probability between a salience network (SN) state and VN state (top right), and significant increases in the transition probability between the SN state and CEN state (bottom right). Follow-up analyses that tested for significant differences between controls and TRD at baseline revealed significantly lower fraction of time spent in the CEN, significantly higher transition probability between SN and VN, and significantly lower transition probability between SN and CEN in TRD compared with controls.

**Fig. 4.**
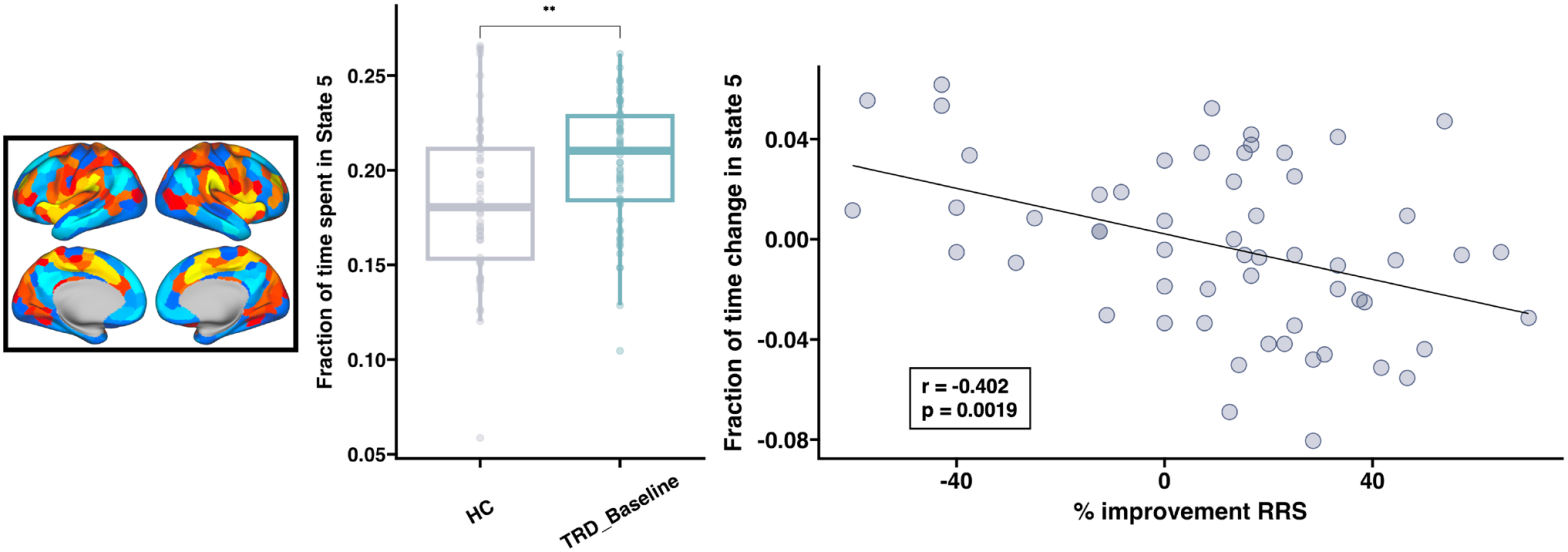
Associations with changes in rumination following ketamine treatment. Percentage improvement in reflective rumination from the RRS measure was significantly correlated with decreases in the fraction of time spent in the salience network (SN) state. As a follow-up, cross-sectional analysis was performed to determine whether trends toward improving symptoms following ketamine treatment moved toward a pattern seen in controls to show TRD participants spent a higher fraction of time in the SN state compared with controls. Scatter plot showing the relationship between SN state dynamics and RRS is displayed on the right, boxplots showing differences between TRD and controls are displayed in the middle, and a visualization of the SN state is displayed on the right. Significant cross-sectional differences imply that FT of CAP 5 in subjects which show greater RRS improvements trend toward FT values seen in HC. *p < 0.05, **p < 0.01, ***p < 0.001.

Following SKI, significant decreases in FT were observed in state 1 (characterized by activity in VN and DAN) (t = -3.57, p = 7.37E-04), and significant increases were observed in state 6 (characterized by activity in CEN) (t = 3.26, p = 1.90E-03). No significant changes in MDT were observed following SKI. Analyses of TP following SKI focused on transitions from all other states to states 1 and 6 to reveal significant increases in TP from state 5 (characterized by activity in SN) to state 6 (t = 3.65, p = 5.79E-04), and significant decreases in TP from state 5 to state 1 (t = -3.04, p = 3.60E-03) (Fig. 3). Decreases in state 5 FT were significantly correlated with improvements in reflective rumination (r = -0.402, p = 1.90E-03) following SKI (Fig. 4), and showed a trending correlation with improvements in brooding rumination that did not survive multiple comparisons correction (r = -0.273, p = 0.0397). Improvements in HDRS were not significantly correlated with changes in CAP metrics. No changes in any of these CAP metrics were observed in the subsample of HCs (who had longitudinal assessments (Supplementary Fig. S3). Finally, results remained similar when based on the CAP states derived from TRD data only as expected since the CAP states themselves did not deviate between all subjects and TRD alone.

Follow-up cross-sectional analyses focused on states and transitions showing significant effects in TRD following SKI, revealed significantly lower FT in state 6 (t = -3.55, p = 5.70E-04), significantly lower TP from state 5 to state 6 (t = -2.44, p = 0.016), significantly higher TP from state 5 to state 1 (t = 3.08, p = 2.60E-03), and significantly higher FT in state 5 (t = 2.932, p = 4.09E-03) in TRD patients at baseline compared with HCs. Furthermore, when comparing TRD post-SKI with HC subjects at baseline to help establish whether changes occurring during ketamine treatment normalize, we found no significant differences in TP from state 5 to state 6 (t = -0.9040, p = 0.3679) and TP from state 5 to state 1 (t =- 0.1612, p = 0.8722) between TRD post-SKI and HC, suggesting these cross-sectional differences in TP significantly trend toward patterns in HCs.

The CAP states derived from clustering data from each group and time point alone and their similarities to the CAP states analyzed here are shown in Supplementary Figure S3. The analysis of framewise similarity in Table 2 suggests, however, that our CAP states are well representative of the entire sample.

## 4 Discussion

To understand how ketamine therapy in depression influences functional brain states, we examined changes in dynamic patterns of BOLD activity in resting-state brain networks pre- to post-serial subanesthetic intravenous ketamine treatment. Across the resting-state fMRI time series and across subjects, CAP analysis revealed six recurring brain states. When examining dynamic changes in brain state co-activation over the course of treatment, we found that ketamine significantly decreased the fraction of time spent in a state with activity in the VN and DAN, and significantly increased the fraction of time spent in a state with activity in the CEN. The fraction of time (FT) can generally be interpreted as the dominance of a particular brain states activity over time, where a long FT implies an overall tendency for a subject to activate a particular brain state, whereas a lower FT implies an aversion toward a particular brain state, where a subject is less likely to activate this state over time. When examining changes in the transitions of these brain states to the other identified brain states pre- to post-treatment, we found that transitions from an SN state to the VN/DAN state significantly decreased, whereas transitions from the SN state to the CEN state significantly increased. Furthermore, we found that decreases in the occurrence of the SN state were significantly associated with improvements in reflective rumination. Together, our results suggest that ketamine treatment modulates dynamics between SN, VN, and CEN functional circuitry, with most of the observed changes seen in patients post-treatment trending toward patterns seen in HCs based on significant cross-sectional differences between TRD and HC at baseline. The analysis of transitions between these functional states suggests that the SN is driving the changes in visual and central executive networks, and the SN is prominently involved in rumination as consistent with prior reports (Lydon-Staley et al., 2019; Menon, 2015; Ordaz et al., 2017).

### 4.1 Visual and dorsal attention networks

Decreases in the fraction of time spent in a state with activation in VN and DAN were observed in TRD following SKI. The VN includes primary and secondary visual association areas (striate and many extrastriate regions), while the DAN includes the frontal intraparietal sulcus and frontal eye fields as well as the middle temporal region and superior parietal lobule and receives important connections from the VN important for orienting and visuospatial attention (Fox et al., 2006; Kincade et al., 2005; Seitzman et al., 2019; Vossel et al., 2014). Although the visual cortex and visual processing are not classically thought of as contributing to MDD, there is increasing and overlooked evidence that the visual cortex plays a role in depression and antidepressant efficacy (Wu et al., 2023). This evidence suggests that MDD patients show impairments in emotional processing within the primary visual cortex, abnormalities in filtering of irrelevant information, and show more autonomy in the ventral and dorsal streams of visual processing (Wu et al., 2023). Our finding also shows correspondence with prior ketamine studies in depression that have examined brain activation or functional connectivity with standard fMRI methods. For example, observations from our own group using fMRI, perfusion (pseudo-continuous arterial spin labeling, pCASL), or diffusion MRI in an overlapping TRD sample have repeatedly identified changes in visual and visual attention systems as being involved in ketamine response. Specifically, we have previously reported increases in VN and DAN cerebral blood flow (Sahib, Loureiro, Vasavada, Kubicki, Joshi, et al., 2020), decreases in VN and DAN BOLD activity related to a response inhibition task (Sahib, Loureiro, Vasavada, Kubicki, Wade, et al., 2020), changes in VN white matter microstructure (Taraku et al., 2023), and increases in habenular–VN functional connectivity (Taraku et al., 2024) following ketamine treatment. Changes in most of these neurobiological markers related to symptomatic improvements post-ketamine treatment. Several independent ketamine studies have also implicated the involvement of visual networks, mostly including secondary visual association regions. For example, Evans et al.(2018) observed increased resting-state functional connectivity between the DMN and VN, and DAN nodes as well as the insula (a key node of the SN) pre- to post-ketamine treatment in MDD. Rivas-Grajales et al. (2021) similarly found increased habenular–VN resting-state connectivity, which is associated with antidepressant response post-ketamine treatment. Further, Reed et al. (2018) reported changes in brain activation for an emotional valence and congruency task in occipital regions that were also associated with antidepressant response in MDD patients receiving ketamine versus placebo. Gilbert et al. (2021) showed that ketamine treatment in TRD accelerated the transmission of GABA and NMDA in the early regions of the visual cortex, and led to inhibition in the early visual cortex and inferior frontal gyrus.

With respect to dynamic changes in functional circuitry, one cross-sectional study showed more functionally optimized, independently organized, and less regulated dorsal and ventral VNs compared in MDD with controls, which included a higher mean variability in dynamic connectivity in MDD (H. Chen et al., 2019). Further in healthy controls, following ketamine administration, ketamine plasma levels were found to have a significant negative influence on dynamic resting-state functional connectivity across VN connections (Spies et al., 2019), adding further evidence that ketamine affects visual and visual–attention network dynamics. Our results together with prior imaging evidence thus suggests that ketamine’s response mechanisms involve a perturbation of connectivity, activation, and dynamics in VN and DAN. These effects are unlikely related to the acute psychomimetic side effects of ketamine, given that these side effects subside 2 hours after administration and post-treatment assessments occurred at least 24 hours after drug administration for the studies cited.

### 4.2 Central executive network

We observed significant increases in the fraction of time spent in the CEN following ketamine treatment. The CEN, composed primarily of the dorsolateral prefrontal cortex (dlPFC) and posterior parietal cortex (PPC), is often considered to be involved in executive function, cognitive tasks, attention, and working memory, and, unlike the DMN, is active during goal-oriented tasks (Mulders et al., 2015). The CEN is also considered to be involved in emotion regulation via strategies such as cognitive reappraisal, which involves top–down regulation of emotion processing regions (Buhle et al., 2014; Morawetz et al., 2017; Rive et al., 2013). The role the CEN plays in cognitive control and emotion regulation has made regions of this network promising targets for antidepressant treatment (Park et al., 2019). Using conventional and dynamic fMRI analysis approaches, the CEN has been widely implicated in depression pathophysiology. For example, prior observations include reports of decreased functional and effective connectivity between the CEN and DMN (Li et al., 2021; Zheng et al., 2015), decreased functional connectivity within regions of the CEN, including the dlPFC and the PPC (Kaiser et al., 2015), and decreased occurrences of a coactive dynamic CEN and DMN state (Dini et al., 2021) in MDD patients compared with controls.

Following ketamine treatment, increased resting-state functional connectivity has been found to occur between the CEN with subcortical limbic structures, including the hippocampus and amygdala, in patients with MDD; early changes in connectivity were shown to relate to clinical improvements (Vasavada et al., 2021). Another study found increased resting-state functional connectivity between the dlPFC (a key node of the CEN) and subgenual anterior cingulate cortex, which showed positive associations with antidepressant response post-ketamine treatment (Gärtner et al., 2019). Increased functional connectivity between frontal nodes of the CEN and the DMN posterior cingulate node was also reported in patients treated with ketamine versus placebo (Evans et al., 2018). However, a study using either a low (0.2 mg/kg) or a standard dose (0.5 mg/kg) of IV ketamine found a reduction in functional connectivity between frontal CEN nodes and the dorsal anterior cingulate at the standard dose, and even more pervasive reductions in connectivity between fronto-parietal CEN nodes at low dose in patients with MDD pre- to post-ketamine treatment where changes were shown to associate with improvements in depressive symptoms and/or suicidality (M.-H. Chen et al., 2019). Taken together, these findings seem to suggest that ketamine serves to decrease connectivity within the CEN, but serves to increase functional connectivity between the CEN and other major resting-state networks including the DMN and limbic regions.

Prior studies investigating the molecular mechanism of ketamine’s antidepressant action have observed increases in glutamate cycling and extracellular glutamate in the prefrontal cortex (PFC) (Abdallah et al., 2016). This is associated with NMDA receptor blockade on GABAergic interneurons, leading to a disinhibition of glutamate and subsequent surge in AMPA receptor activity in the PFC, which ultimately facilitates post-synaptic neuroplasticity signaling pathways (Abdallah et al., 2016). In the context of this neurobiological model, it is likely that the increase in PFC glutamate and neuroplasticity results in greater and more frequent activity in the PFC, which facilitates frequent co-activation of networks involving PFC regions, including the CEN. Thus, this signaling cascade from NMDA receptor blockage likely results in more frequent co-activation of networks involving the PFC, thus leading to greater dominance of executive control networks during rest.

Disruptions in CEN circuitry may also contribute to cognitive deficits commonly seen in depression (Disner et al., 2011), such as executive function and working memory impairments (Rose & Ebmeier, 2006; Snyder, 2013). Subanesthetic ketamine treatment in MDD has been shown to enhance aspects of neurocognitive function including in the domains of working memory, processing speed, and episodic memory (Zavaliangos-Petropulu, McClintock, et al., 2023), which suggests that ketamine may affect systems involved in both the depressive and cognitive deficits associated with MDD. These findings together with our observations suggest that ketamine treatment alleviates MDD-related within-network CEN hypoactivity, which may affect a range of deficits associated with depression. Whether ketamine-induced CEN changes lead to downstream effects contributing to improvements in cognitive and emotional control remains a goal for future study.

### 4.3 Transitions between the salience network and central executive and visual networks

Our analysis of state transitions revealed significant increases in the transition probability from the salience network state to the CEN state, as well as significant decreases in the transition probability from the salience network state to visual network state. The salience network, consisting of nodes primarily in the anterior insula and dorsal anterior cingulate cortex, is believed to play a role in subjective salience, emotional judgment, and facilitates a causal role in the dynamic switching between the DMN and CEN, thus also playing a bottom-up role in attentional control (Menon & Uddin, 2010). The anterior insula node of the salience network has been described as a gatekeeper of executive control due to its role in orchestrating brain network dynamics (Molnar-Szakacs & Uddin, 2022; Uddin, 2015). It is also believed to play a role in emotional processing and emotional control through extensive subcortical connections (Mulders et al., 2015). The salience network, along with the DMN and CEN, together comprises a set of large-scale networks which encompass the triple network model, which posits that dynamic interactions between these networks underlie a wide range of psychopathologies (Menon, 2011). Prior studies investigating resting-state networks in MDD have found abnormalities between the SN and DMN, including hypoconnectivty between SN and DMN (H. Chen et al., 2019; Kaiser et al., 2015), as well as increased dynamic FC fluctuations between SN and DMN (Kaiser et al., 2016). Since our findings revealed increased occurrences of CEN states and decreased occurrences of VN states, our follow-up analyses of transition probability imply that the SN is driving these changes following ketamine treatment. Partially in line with our findings, another fMRI study of ketamine in MDD showed a normalization of connectivity between the SN with the DMN and CEN, to support the triple network dysfunction model of MDD and its modulation by ketamine. Together these findings might suggest that ketamine treatment targets SN-related circuitry, which in turn modulates broader brain circuitry related to emotional, cognitive, and sensory processing.

### 4.4 Associations with rumination

When examining the association between changes in brain state dynamics and improvements in clinical symptoms, we found a significant association between decreases in the occurrences of an SN state and improvements in reflective rumination, though did not find significant associations for overall mood scales. Rumination is a prominent symptom of depression, characterized by repetitive and passive self-focused thoughts on one’s negative feelings and symptoms of distress (Parola et al., 2017). However, recent theories suggest rumination is a multidimensional construct consisting of two distinct components, which include brooding, considered maladaptive and links to depressive symptoms, and reflection, which is “a purposeful turning inward to engage in cognitive problem-solving to alleviate one’s depressive symptoms” (Parola et al., 2017). Although prior research on rumination has focused primarily on brooding in depression, one study found that after performing a factor analysis on RRS scores, a distinction between reflection and brooding could only be found in formerly depressed and never depressed individuals, but did not obtain this distinction in currently depressed individuals. Furthermore, this study identified another factor in depressed individuals which they labeled “intentional rumination” (Whitmer & Gotlib, 2011). Therefore, the precise interpretation of our finding that reflective rumination is reduced following SKI and how this is distinguished from brooding is yet to be determined, since rumination may be more complex in depressed individuals than previously theorized. Further research is needed to establish how ketamine treatment alters the dimensions of rumination in depressed individuals.

The SN is involved in responding to externally salient cues, and acts as a dynamic switch between the DMN involved in negative, self-referential processing, and the CEN, involved in executive function. Rumination has often been associated with dysfunction in the DMN (H.-X. Zhou et al., 2020). Many studies have also linked the SN and intrinsic connectivity between nodes of the SN and DMN with rumination (Burrows et al., 2017; Ordaz et al., 2017), including in MDD specifically (Cooney et al., 2010; Paul et al., 2013; Ray et al., 2005). Furthermore, one particular study examining the topological features of large scale brain networks found that the SN is expanded nearly two-fold in patients with depression (Lynch et al., 2024). At the same time, previous studies have suggested that overactivity of the SN relates to abnormal affective processing (Schimmelpfennig et al., 2023), including negative response bias to affective stimuli in MDD (Hamilton et al., 2013). Since our results show that time spent in the CEN is significantly greater after SKI, and that transition probability from SN to CEN increased, it is possible that less time spent in the SN state may reflect a restoration of the regulation of negative emotional states, as a consequence of more time spent in the CEN. These findings align with prior CAP findings of increased dwell time in a state composed of co-active SN and DMN as well as increased transitions between SN–DMN states to prototypical DMN states, which both associate with greater rumination in depression (Kaiser et al., 2019).

We note that although significant improvements in HDRS occurred following SKI, we did not observe significant correlations between HDRS changes and changes in CAP metrics over time. While correlations between brain changes and changes in clinical measures can provide insight into potential neurobiological mechanisms that induce symptom improvements, it is possible that the measured brain changes here only describe certain brain–symptom relationships, given the distributed pathophysiology of MDD (Buch & Liston, 2020). In this case, while ruminative symptoms in this study as well as several previous studies (Belleau et al., 2022; Kaiser et al., 2019; C. Liu et al., 2023) are correlated with CAP dynamics, it is possible that the neurobiological mechanisms underlying overall depressive symptoms measured by HDRS may have distinct neurobiological drivers independent of CAP dynamics. For example, it may be the case that connectivity in particular functional circuits are correlates of HDRS symptom improvements rather than functional dynamics across the whole brain. It is also possible that more statistical power is needed to identify relationships between CAP dynamics and HDRS changes, which would imply that the link between these symptoms and CAP dynamics is weaker than that of rumination and CAP dynamics.

### 4.5 Cross-sectional differences with controls

By performing post hoc cross-sectional analyses focusing on the dynamic states showing significant effects following ketamine treatment, we found significant differences between TRD and HC at baseline in the occurrences of a CEN state, SN state, and the transitions from a SN state to both the CEN and VN state. Comparisons between TRD–post-SKI and HCs suggest that most of the observed changes following ketamine treatment normalize aberrant network dynamics present in depression, with patterns showing more similarity to HCs following treatment. These cross-sectional findings are also supported by prior research which has found differences in CEN (Kaiser et al., 2015; C. Liu et al., 2023), SN (Manoliu et al., 2014), and VN (H. Chen et al., 2019) between depressed individuals and controls.

### 4.6 Limitations

We acknowledge several limitations of this investigation. We note that this study was not a randomized clinical trial and did not include a placebo control condition. However, this study was designed as a mechanistic study focused on imaging outcomes and delineating relevant neural circuitry, rather than on the efficacy of SKI. We also note that patient participants were allowed to continue concurrent stable antidepressant medication. However, patients served as their own controls in longitudinal analyses, with our study focusing on within-subject changes. The antidepressant effects of ketamine are reported to peak at around 24 hours post-infusion and to be greater after serial therapy (Marcantoni et al., 2020). However, the temporal relationships between brain circuit-level neuroplasticity and therapeutic response may be more complex and the timing of post-treatment assessments may influence results.

We also acknowledge that there are limitations associated with the CAP method used here, which includes the identification of specific clusters used to identify brain states, which can sometimes be arbitrarily defined and differ across studies. However, despite brain states not always being identical across studies, the brain states identified here show correspondence with commonly identified and frequently studied resting-state brain networks (Uddin et al., 2019), overlap with states identified in prior studies (Belleau et al., 2022), and our findings regarding the dynamics of the CEN also show correspondence with prior CAP studies (Kaiser et al., 2019; C. Liu et al., 2023). Furthermore, we show that when using a different number of clusters (k-1 and k+1), we find similar brain states and also show similar significant results with k-1 clusters, suggesting that the dynamic activity patterns we are seeing are not contingent on one configuration of clusters. One limitation with this approach was that we could not replicate any results using k+1 clusters, suggesting that the observed changes in brain dynamics cannot be identified when using a set of seven states. Although CAP analysis requires a discrete number of brain states to analyze brain dynamics, other studies have modeled brain dynamics as a smooth manifold of brain activity patterns (Rué-Queralt et al., 2021). Thus, a discrete number of brain states may be an approximation to the landscape of brain dynamics, and future research should reconcile findings from CAP studies with other methods to estimate brain dynamics.

In addition to these points, we acknowledge that we observed minor differences in the spatial patterns of CAP states generated from HC alone compared with our entire sample. However, we note that our aim was not to identify differences in CAP states across groups but rather to examine differences in CAP dynamics between a common set of states across groups and treatment. Additionally, our analysis of subject-level similarity to cluster centroids in Table 2 suggests that each group of subject and time points does not significantly differ in their spatial patterns to each cluster centroid, implying that these CAP states are well representative of the entire sample. Further research is needed to examine how CAP states differ across diagnostic groups and how this relates to differences in CAP dynamics and static FC. Lastly, in a supplementary analysis, we show that these CAP metrics are relatively stable across controls scanned twice without intervention, suggesting that these observed changes likely represent brain changes induced by ketamine treatment.

### 4.7 Conclusions

Serial ketamine treatment produces changes in resting-state functional dynamics of large-scale brain networks including the CEN, VN/DAN, and SN. Changes in the SN in particular relate to improvements in rumination symptoms. The observed changes induced by ketamine treatment also normalize aberrant patterns of brain network dynamics seen in depressed patients when compared with healthy controls. These findings provide further insight into the functional brain systems-level mechanisms accounting for the therapeutic effects of ketamine in MDD.

## Supplementary Material

Supplementary Material

## Data Availability

The data used in this study are publicly available in the NIMH Data Archive in collection 2844 at https://nda.nih.gov/edit_collection.html?id=2844. Code used to run analyses is available on GitHub at https://github.com/btaraku/CAP_analysis.
