# Supplementary material for: Modulation of functional network co-activation pattern dynamics following ketamine treatment in major depression
Source: Imaging Neurosci (Camb). 2025 Oct 15;3:IMAG.a.936. doi: 10.1162/IMAG.a.936 (PMC12529346; doi:10.1162/IMAG.a.936)
Supplement: Supplementary Material [file IMAG.a.936_supp.pdf]

Supplementary Materials

Elbow criterion to determine the optimal number of clusters:

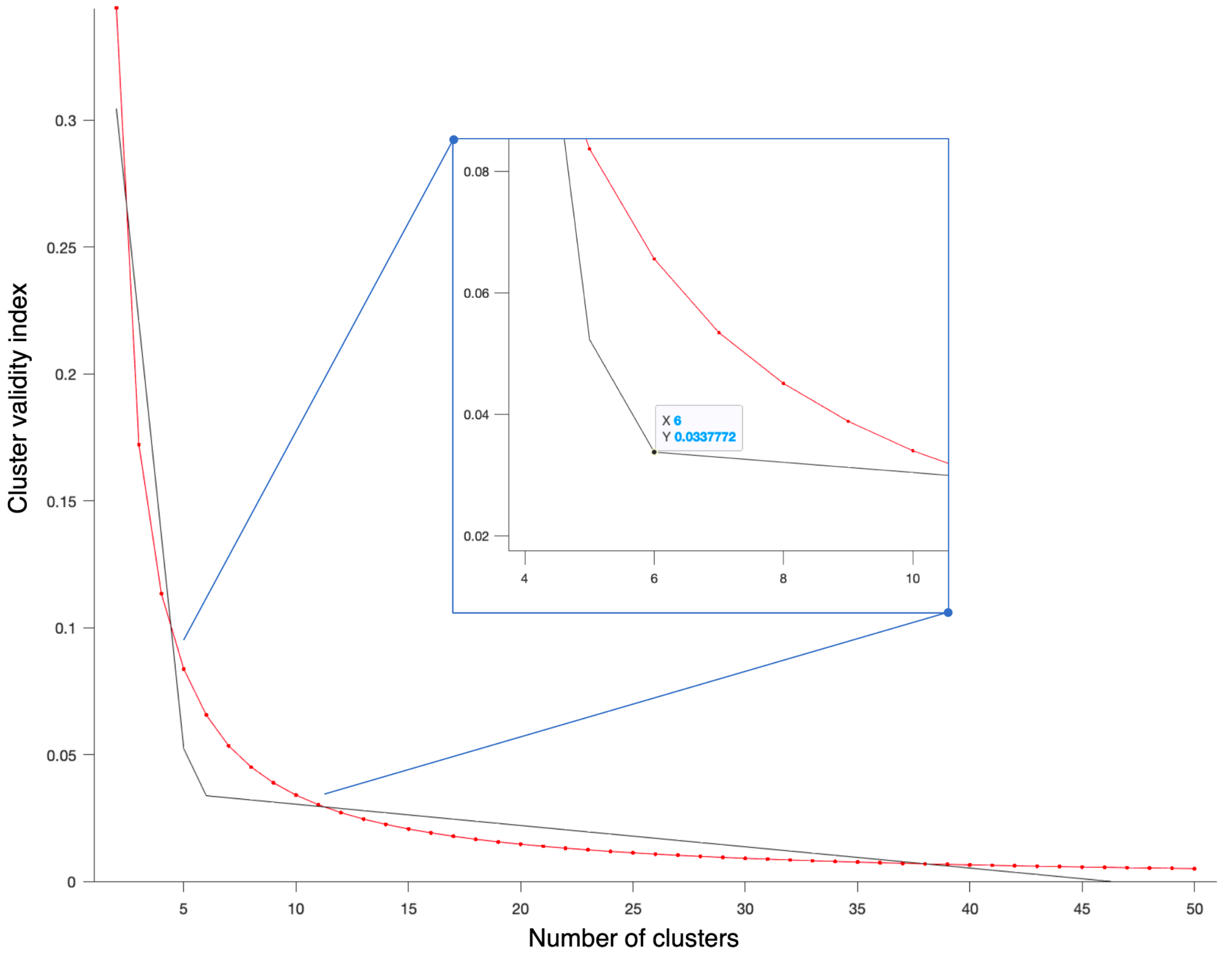

Supplementary Figure 1: Determination of number of clusters. Since the elbow criterion may involve a subjective determination of a sharp point along a smooth curve of points, we followed an approach to determine a single location of the elbow point. Here, we used an L-curve made up of 3 distinct slopes (shown in black), and fit this to a set of points containing the cluster validity index for each number of clusters used (shown in red) using least squares regression. The fitted curve then gives 2 distinct points where the slope suddenly changes, and we use the point where the magnitude of the slope reaches its minimum, indicating a number of clusters where the rate of decrease in error slows down as we add more clusters. This approach revealed 6 clusters to be optimal. An expanded view of the elbow point is shown in the blue box in the center of the plot.

Silhouette analysis to determine the optimal number of clusters:

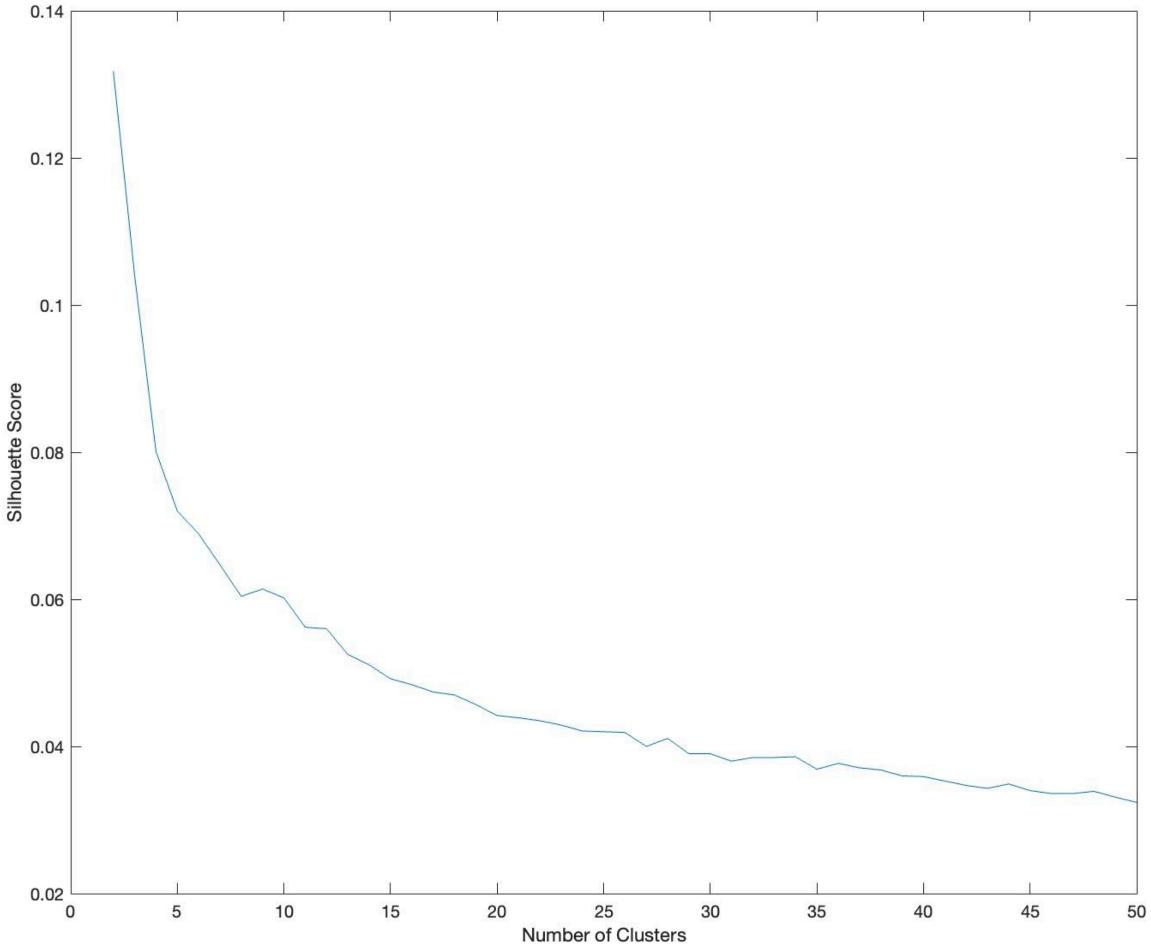

Supplementary Figure 2: Determination of the optimal number of clusters using Silhouette Scores. The silhouette score measures the degree of how similar a point is to its own cluster relative to other clusters, with values ranging from -1 to 1 and with higher scores indicating greater similarity to its own cluster(“Silhouettes: A Graphical Aid to the Interpretation and Validation of Cluster Analysis,” 1987). Based on this, the optimal number of clusters would be k = 2, and since the plot is generally decreasing, a smaller value of k tends to always be more optimal. However, this leads to a non trivial decision, since most CAP studies use a higher number of clusters (often between 5 and 10 brain states) and 2 brain states is likely not enough to fully capture the dynamics of resting state networks across the brain. Furthermore, the silhouette score may be more optimal for convex shaped clusters and not clusters with irregular shapes and varying sizes, and it also may be difficult to achieve high silhouette scores with high dimensional data(“A Deep Density Based and Self-Determining Clustering Approach to Label Unknown Traffic,” 2022). Therefore, we opted to use the elbow criterion on the cluster validity

index to determine the optimal number of clusters, since silhouette scores did not seem to produce an appropriate result for our CAP analysis.

CAP states derived from individual groups and timepoints:

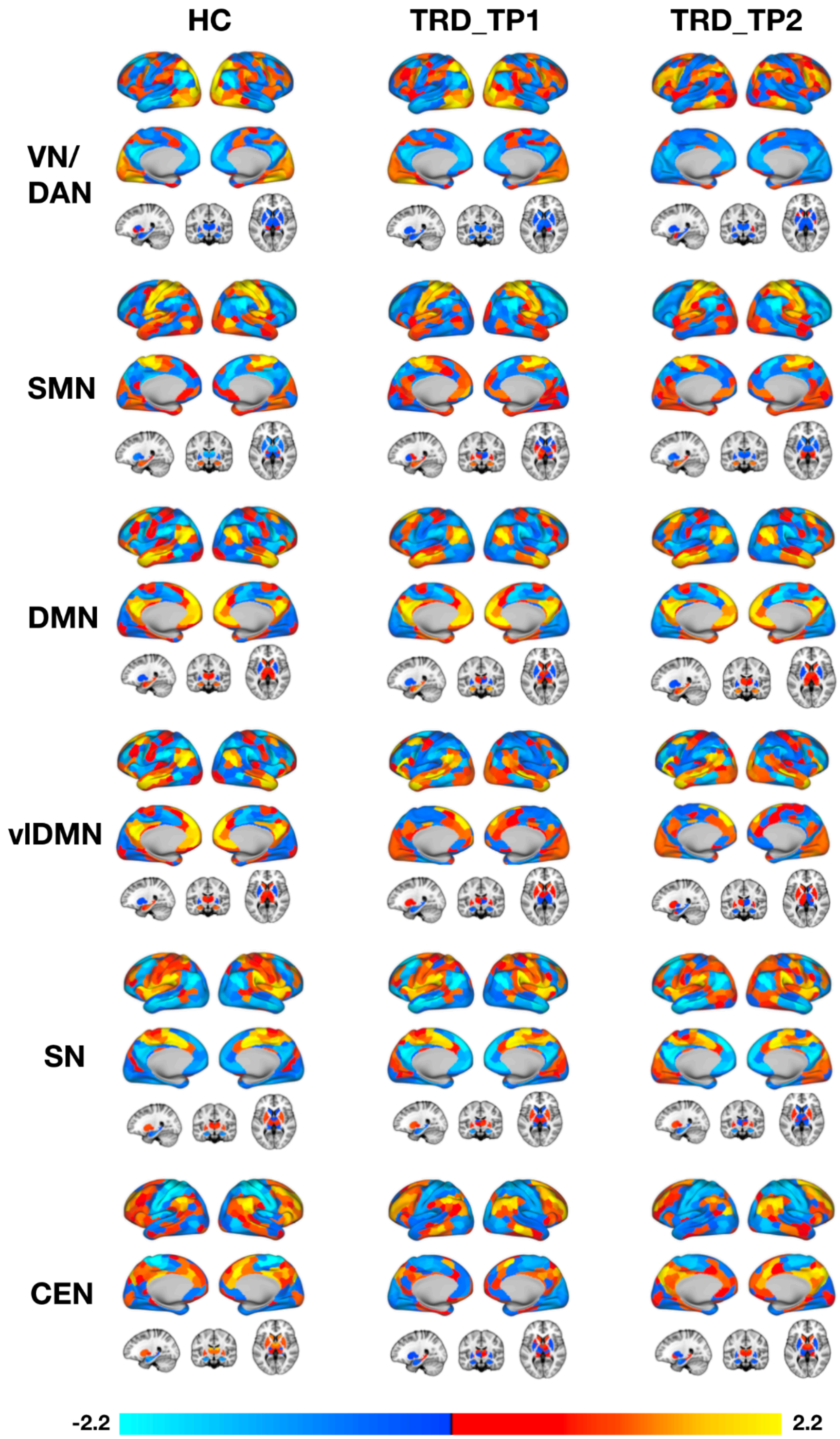

All subjects and timepoints (n=190)	HC Baseline (n=56)	TRD Baseline (n=58)	TRD Post SKI (n=58)
**State 1 (VN/DAN state)**	r = 0.9109<br>(State 6: VN/DAN)	r = 0.9705<br>(State 5: VN/DAN)	r = 0.6923<br>(State 4: DAN/VN)

**State 2 (SMN)**	r = 0.9743<br>(State 5: SMN)	r = 0.9553<br>(State 6: SMN)	r = 0.9772<br>(State 5: SMN/VN)
**State 3 (DMN)**	r = 0.9079<br>(State 1: DMN)	r = 0.9947<br>(State 4: DMN)	r = 0.9820<br>(State 6: DMN)
**State 4 (vlDMN/VN)**	r = 0.6391<br>(State 1: DMN)	r = 0.9850<br>(State 3: vlDMN/VN)	r = 0.9783<br>(State 3: vlDMN/VN)
**State 5 (SN)**	r = 0.9434<br>(State 2: SN)	r = 0.9862<br>(State 1: SN)	r = 0.9798<br>(State 1: SN)
**State 6 (CEN/DMN)**	r = 0.8378<br>(State 3: CEN/DMN)	r = 0.9806<br>(State 2: CEN/DMN)	r = 0.8906<br>(State 2: CEN/DMN)

Supplementary Figure 3: Comparison of CAP states estimated across the whole sample and within each group. In order to determine if the brain states obtained from clustering all the data together were also representative of brain states for each timepoint of TRD and the HC group, we re-ran k-means clustering with 6 clusters on each of these groups independently. Top panel shows a visualization of each state, with the rows corresponding to the states from the entire sample, and the columns corresponding to the most similar CAP state estimated from each group alone. In the bottom panel, the rows of the table correspond to each state determined from the entire sample, which are the same brain states used in the primary analyses of this manuscript, whereas each column corresponds to the groups of subjects that were clustered separately (healthy controls, TRD at baseline and TRD post treatment). Each entry in the table lists the highest spatial correlation (r-value) between the brain state in the corresponding row to the brain states from the subjects clustered in the corresponding column, as well as the brain state number and a network description in parentheses. The brain states from the subjects clustered in each column are arbitrarily numbered, but are included to indicate whether each state maps onto a unique state generated from the entire sample, or if there are multiple states that map onto one state when comparing across the runs of k-means. For TRD subjects at baseline, we found highly similar states (all $r > 0.95$) and with each brain state from all subjects mapping onto a unique brain state from the TRD group alone. Following SKI, we also find highly similar clusters, although not as highly similar with those from the TRD at baseline, but we still find that all brain states determined from clustering all subjects map onto a unique brain state from the post-SKI TRD group. We note that although we see a large decrease in similarity between the VN CAP states, this could be a consequence of less time spent in the VN state, as observed in **Figure 3**, which could affect the data points used in identifying cluster centroids. Likewise, the significant change in time spent in the CEN CAP state for TRD following SKI could also impact the changes in spatial correlation between the two columns of the last row. For the HC group, while we find that most brain states show high similarity within this group, not all of the states determined from all subjects combined map onto unique states from the HC group. In

particular, we find that State 3 and 4 (representing 2 DMN states) from all subjects (see **Figure 3**) both map onto one state from the HC group, suggesting that the HC group only has one DMN state. On the other hand, the HC group showed 2 CEN states, which both showed high spatial similarity to State 6 from all subjects (HC State 4: r = 0.7301). Despite these differences in CAP states, we note that the HC group was only used to make comparisons between TRD with the states that showed significant changes following SKI, and that states 3 and 4 were not used to make comparisons between HC and TRD. We also note that the clusters used in the primary analysis incorporated many more data points when all subjects are combined, so any deviations in brain states from clustering individual groups separately could also be the result of lower statistical power.

Longitudinal healthy control analysis:

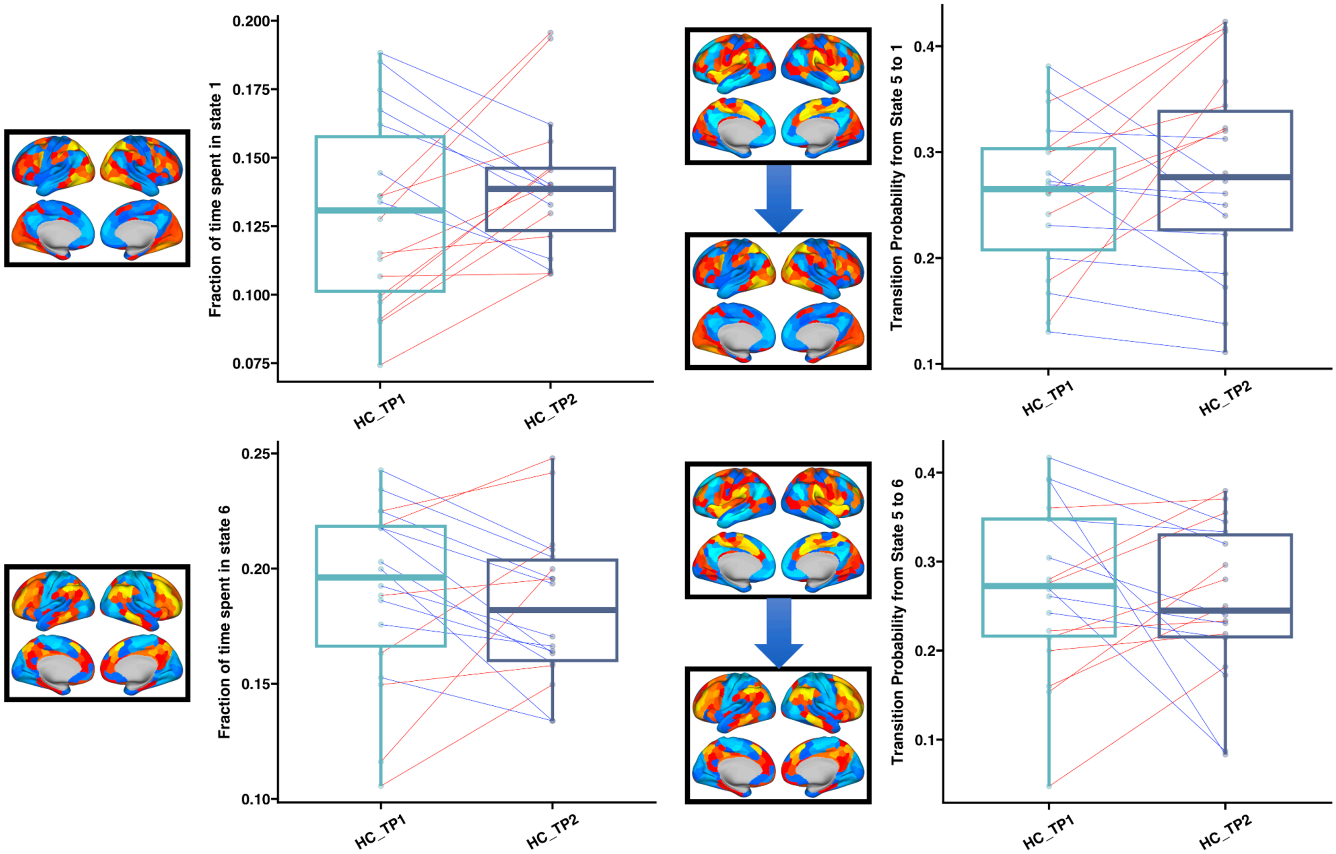

CAP measure	t-statistic	p-value
**TF State 1**	1.0763	0.297
**TF State 6**	-0.709	0.488
**TP State 5 to 1**	1.0443	0.311

**TP State 5 to 6**	-0.0753	0.56

Supplementary Figure 4: Follow-up analysis of longitudinal healthy controls (HC). In order to ensure the significant effects we observe are the result of ketamine treatment and not due to some change in the scanning environment, we conducted paired t-tests using our subsample of controls with two timepoints, scanned over a similar time period to the subjects receiving ketamine treatment (2 weeks). Age and sex did not significantly differ in this longitudinal subsample compared to the HC subjects without longitudinal scans using independent samples t-tests and chi-square tests ($t = 1.4128$, $p = 0.1634$) ($\chi2 = 0.0273$, $p = 0.8688$). We did not observe any significant changes in this sample when testing the states and transitions showing significant changes in our depressed cohort, suggesting these changes are due to ketamine treatment. Box Plots and spaghetti plots are shown across the different states and transitions above, and below are the respective t-statistics and p-values.

CAP states without GSR:

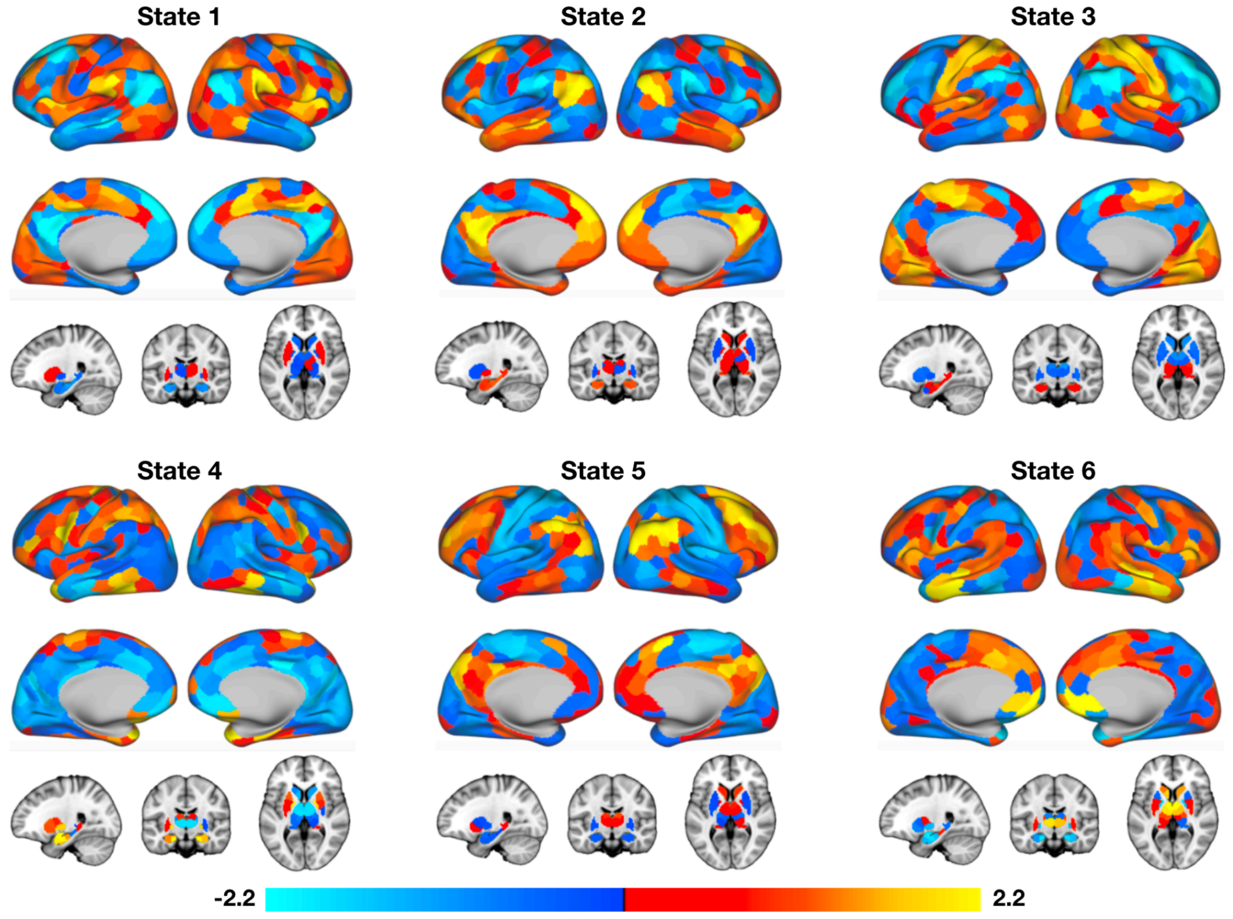

	VN	SMN	DAN	SN	LIMBIC	CEN	DMN
**cluster 1**	0.1522	0.0668	0.3286	0.4691	-0.1597	-0.0343	-0.6823
**cluster 2**	-0.1253	-0.0712	-0.3766	-0.4223	0.1513	-0.013	0.7068
**cluster 3**	0.2996	0.6076	-0.0971	-0.0556	-0.0935	-0.5685	-0.2006
**cluster 4**	-0.2091	0.1888	0.3567	-0.1057	0.1716	-0.0105	-0.281
**cluster 5**	-0.1694	-0.5925	0.086	-0.0821	6.06E-04	0.5773	0.2366
**cluster 6**	-0.0343	-0.1191	-0.3474	0.171	-0.0079	0.0297	0.2553

	Cluster 1	Cluster 2	Cluster 3	Cluster 4	Cluster 5	Cluster 6
**mean FD**	0.1517	0.1425	0.1556	0.1361	0.1442	0.1538
**std FD**	0.1804	0.1524	0.18	0.1488	0.1487	0.1616

Supplementary Figure 5: CAP states obtained without using global signal regression (GSR). There is an ongoing debate concerning whether GSR should be included as a denoising process for fMRI since the global signal can contain relevant neurological information while at the same time reflect global artifacts such as from head motion (Liu et al., 2018). Given the lack of consensus of whether GSR should be implemented, we performed a supplemental clustering analysis using the same processing steps but without using GSR. Here, we also found k=6 to be an optimal cluster number, but with slight spatial deviations in functional states. The states are visually shown in the top panel, the middle panel shows spatial correlations between each CAP state and each of the 7 network atlases from the Schaefer atlas, as in Figure 2 of the manuscript, and the bottom panel shows the mean and standard deviation of framewise displacement (FD) in each cluster. From the middle panel, we see that there is generally less similarity between these CAP states and known resting state network atlases (in the VN, DAN, SMN, SN, and CEN) than the CAP states acquired with GSR (Figure 2). Notably, there does not appear to be a clear VN or DAN (implicated in visuo-spatial attention) state, unlike in our GSR states, which are of clinical relevance given that prior studies have frequently found changes in visual networks following ketamine treatment. In the bottom panel, there is still generally low FD across these clusters, suggesting they are likely not capturing major motion states. However, the mean and std FD across these states does appear to be less consistent across states, with clusters generally showing both higher and lower mean FD across the clusters than compared to the results with GSR, which show highly consistent mean FD throughout all states. Ultimately, we chose to use our results with GSR, considering that the states obtained show higher correspondence with known resting state networks, which are of clinical importance.

Effect of cluster number on CAP results:

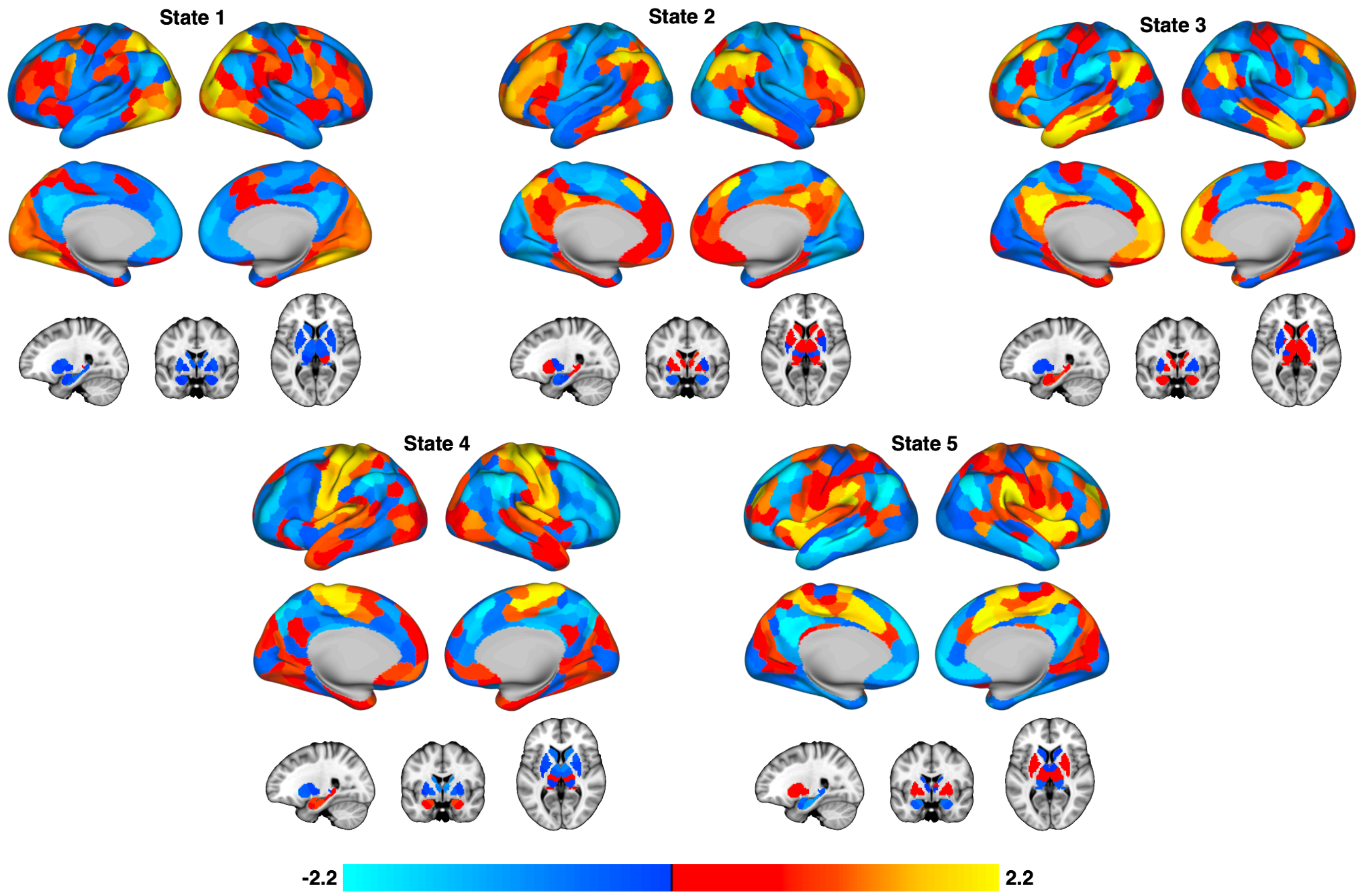

Spatial correlations between CAP states				k=5 CAP state group statistics		
	k=6 state 1	k=6 state 5	k=6 state 6	CAP measure	t-statistic/ r-value	p-value
**k=5 state 1**	**0.9742**	0.335	-0.0509	**TF State 1**	**t=-4.248**	**8.05E-05**
**k=5 state 2**	0.0091	-0.4087	**0.9806**	**TF State 2**	**t=2.91**	**5.20E-03**
**k=5 state 3**	-0.669	-0.8652	0.151	**TP State 5 to 1**	t=-1.84	0.07
**k=5 state 4**	-0.2141	0.1134	-0.9297	**TP State 5 to 2**	**t=3.47**	**9.86E-04**
**k=5 state 5**	0.2114	**0.9964**	-0.2654	**corr(RRS,TP state 5)**	**r=0.348**	**8.1E-03**

Supplementary Figure 5: CAP analysis using $k_{optimal}$-1 clusters. CAP analysis was performed on the same group of subjects using the same metrics, instead using 5 clusters. We found that the clusters were quite similar to the optimal 6 cluster solution based on visual inspection, however, we found only one DMN state here unlike the 6 cluster solution. Using the states that showed significant effects of treatment from the 6 cluster solution, we computed Pearson correlation coefficients between each of these states and all states from the 5 cluster solution, indicated in the table on the bottom left.

We found states with high spatial similarity (bolded and italicized entries in table) and used these to run the same group level statistics to examine treatment effects of ketamine in our TRD sample. Here, we found significant effects in 4 out of the 5 tests, suggesting that the changes in state dynamics we observed is not entirely contingent on the 6 cluster solution, and can be mostly replicated using 5 clusters as well.

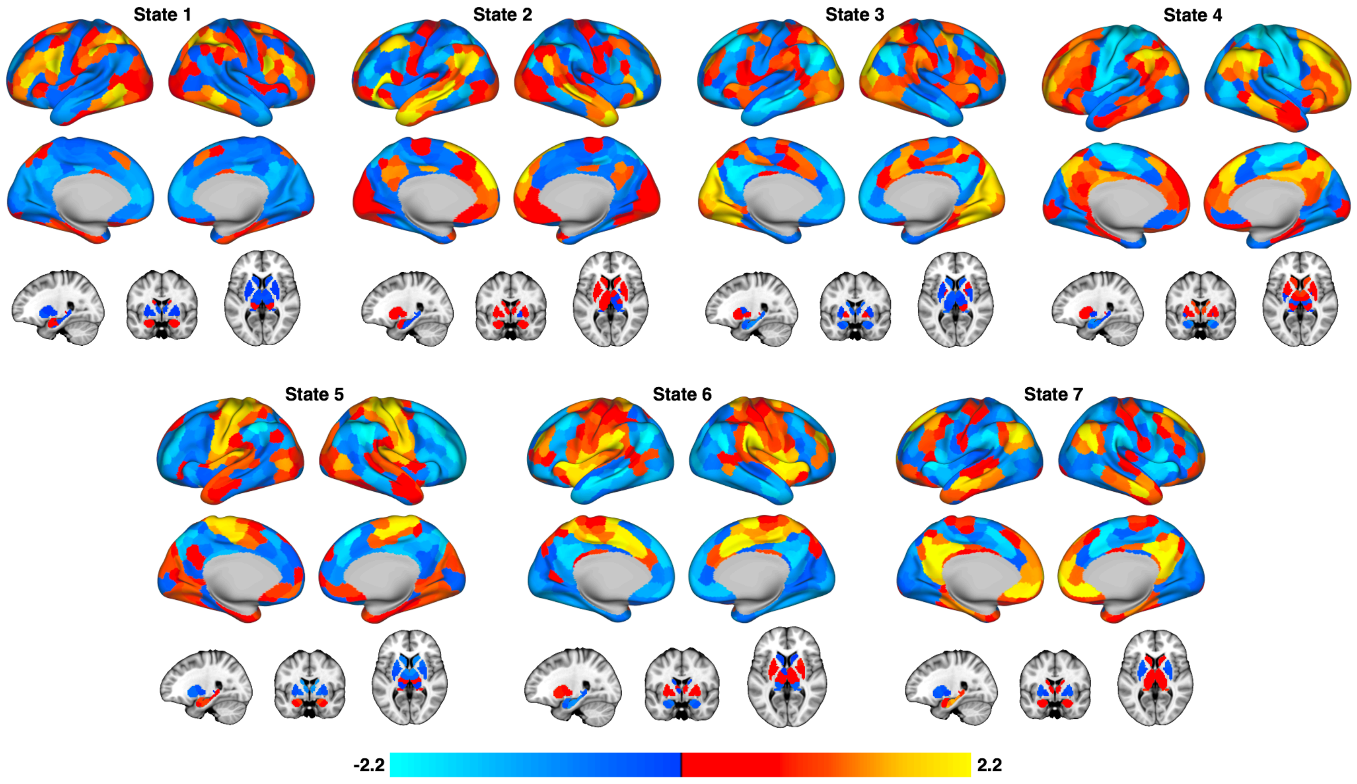

Spatial correlations between CAP states				k=7 CAP state group statistics		
	k=6 state 1	k=6 state 5	k=6 state 6	CAP measure	t-statistic/ r-value	p-value
**k=7 state 1**	0.7249	0.0582	0.3729	**TF State 3**	t=-2.327	2.36E-02
**k=7 state 2**	-0.5976	-0.5223	-0.1429	**TF State 4**	t=1.5233	1.33E-01
**k=7 state 3**	**0.7367**	0.6935	-0.283	**TP State 6 to 3**	t=0.809	4.22E-01
**k=7 state 4**	-0.0332	-0.3605	**0.9664**	**TP State 6 to 4**	t=0.916	3.63E-01
**k=7 state 5**	-0.1143	0.1573	-0.9528	**corr(RRS,TF State 6)**	r=0.234	7.97E-02
**k=7 state 6**	-0.0148	**0.9292**	-0.2418			
**k=7 state 7**	-0.544	-0.8821	0.2769			

Supplementary Figure 6: CAP analysis using $k_{optimal}+1$ clusters. CAP analysis was performed on the same group of subjects using the same metrics, instead using 7 clusters. We also found similar CAP states to the states identified with the optimal clustering solution, however, we note that certain networks from the 6 cluster solution were fragmented into several distinct clusters when using 7 clusters. We computed the spatial similarity between clusters showing significant effects from the 6 cluster solution, and found states with high similarity (bolded and italicized entries in table), but not as high as the spatial similarity between the states from the 5 and 6 cluster solutions. For state 1 from the 6 cluster solution, we note that both state 1 and 3 from the 7 cluster solution showed a high degree of similarity with very similar r-values, which seems to imply that this state was fragmented into 2 states when using 7 clusters. Additionally, although we found a high degree of spatial similarity between state 6 from the 6 cluster solution and state 4, we also note there is a moderate similarity with state 1, and based on visual inspection shows strong activation in prominent regions of the central executive network (CEN), suggesting the CEN may also be fragmented into different clusters when using the 7 cluster solution. We opted to use the clusters with the highest spatial similarity with the clusters determined to be optimal, but did not find significant changes in our TRD sample when using 7 clusters. However, since brain states representing prominent networks of interest appear to be fragmented across different states using 7 clusters, this suggests that using 5 or 6 clusters may preserve relevant networks in these brain states and therefore allow us to better examine dynamic changes between these states.

Static Functional Connectivity Analysis:

**ROI_1**	**ROI_2**	**t-stat**	**p-value**
7Networks_LH_Vis_23	7Networks_LH_Vis_28	-6.7331	8.85E-09
7Networks_LH_Vis_27	7Networks_LH_Vis_28	-6.118	9.21E-08
7Networks_LH_Vis_28	7Networks_RH_Vis_22	-6.4438	2.67E-08
7Networks_LH_Vis_28	7Networks_RH_Vis_27	-6.0015	1.43E-07
7Networks_LH_Default_PFC_1	7Networks_LH_Default_pCunPCC_8	-6.1196	9.16E-08

Supplementary Table 1: To understand how dynamic FC results using CAP analysis compared to traditional static FC measures, we conducted a static FC analysis using the same sample of TRD subjects by investigating longitudinal changes in static FC following SKI, using paired t-tests. Here, we computed static FC by computing the Pearson correlation coefficient between all nodes of the same set of ROIs (Schaefer

cortical and Tian subcortical atlas) for each subject. We corrected for multiple comparisons across all unique ROI to ROI connections using Bonferroni correction (number of unique connections in symmetric FC matrix = (nROI*(nROI - 1))/2). Following SKI, we found decreases in static FC primarily between nodes of the VN across both hemispheres, as well as with nodes of the DMN. These regions are of relevance since we identified changes in FT of the VN and DAN CAP following SKI, which may be related to decreases in static FC between several nodes comprising activity in this CAP state. We also note that there was an observed decrease in spatial similarity between this CAP and a VN CAP state acquired from clustering only TRD post-SKI (Table X), which could also be explained by significant decreases in static FC between nodes of this state. Significant regions are listed in the first 2 columns of the table above, where entries in each row correspond to a significant change in static FC between the regions in ROI_1 and ROI_2. It should be noted that these connections are undirected, so the ordering of ROI_1 and ROI_2 is arbitrary and can be swapped.

References

A deep density based and self-determining clustering approach to label unknown traffic. (2022). Journal of Network and Computer Applications, 207, 103513.

Liu, X., Zhang, N., Chang, C., & Duyn, J. H. (2018). Co-activation patterns in resting-state fMRI signals. NeuroImage, 180(Pt B), 485–494.

Silhouettes: A graphical aid to the interpretation and validation of cluster analysis. (1987). Journal of Computational and Applied Mathematics, 20, 53–65.
